# Saturated fat exacerbates mitochondrial dysfunction through remodelling of ATP production and inflammation in Barrett’s oesophagus compared to monounsaturated fat, particularly in contrast to oesophageal adenocarcinoma

**DOI:** 10.1016/j.neo.2025.101173

**Published:** 2025-05-16

**Authors:** Kathleen A.J. Mitchelson, Fiona O’Connell, Kieran Wynne, David Matallanas, Jacintha O’Sullivan, Helen M. Roche

**Affiliations:** a Nutrigenomics Research Group, UCD Institute of Food and Health, and School of Public Health, Physiotherapy and Sports Science, University College Dublin, Dublin, Ireland; b UCD Conway Institute, University College Dublin, Dublin, Ireland; c Department of Surgery, Trinity St. James’s Cancer Institute and Trinity Translational Medicine Institute, St. James’s Hospital and Trinity College Dublin, Dublin, Ireland; d Systems Biology Ireland, University College Dublin, Dublin, Ireland; e School of Medicine, University College Dublin, Dublin, Ireland; f Institute for Global Food Security, School of Biological Sciences, Queens University Belfast, Belfast, United Kingdom

**Keywords:** Obesity, Diet, Saturated fatty acids, Monounsaturated fatty acids, Barrett’s oesophagus, Oesophageal adenocarcinoma, Cancer, Metabolism, Inflammation

## Abstract

Obesity-related oesophageal adenocarcinoma (OAC), arising from Barrett’s oesophagus (BO), incidence rates are rising coincident with high-fat diets. However, adipose tissue phenotype drives metabolic characteristics. Prior feeding studies demonstrated that obesogenic diets enriched in saturated fatty acids (SFA) induce a more adverse metabolic and pro-inflammatory adipose phenotype, compared to monounsaturated fatty acids (MUFA) enriched high-fat diets, despite equal obesity. We hypothesise that different fatty acids may alter the progression of BO to OAC, wherein SFA may be more pathogenic compared to MUFA. Proteomic analysis shows that SFA, not MUFA, increases fatty acid metabolism, oncogenic signalling, and mitochondrial respiratory chain to a greater extent in BO but not in OAC cells. Cellular metabolic analysis validated proteomic findings to show mitochondrial dysfunction in BO but showed an increase in glycolysis in OAC following SFA treatment compared to MUFA. Additionally, it showed a decrease in mitochondrial ATP production following treatment of SFA in BO and OAC cells. Reduction of SFA intake may be beneficial as a supplementary treatment approach to manage and/or prevent OAC progression.

## Introduction

Obesity has overtaken smoking as the leading cause of cancer and is strongly associated with increased risk of 13 different cancers, including a range of gastrointestinal cancers such as oesophageal, stomach, and colorectal [1,2]. Abdominal obesity, reflecting a preponderance of visceral adipose tissue (VAT) has long been associated with oesophageal adenocarcinoma (OAC) [3]. This could be attributed to mechanical elements wherein VAT increases pressure around the stomach resulting in prolonged acid exposure resulting in reflux-induced Barrett’s oesophagus (BO), a pre-malignant condition that often precedes OAC [4,5]. Furthermore, visceral adiposity is associated with progression from BO to high-grade dysplasia and OAC [6,7]. Remarkably, in OAC, viscerally obese and metabolic dysfunction patients displayed increased oxidative phosphorylation, inflammation, and metabolites associated with driving tumorigenesis. Specifically, there was an increase in eotaxin-3, IL-17A, IL-17D, IL-3, MCP-1, and MDC levels paired with altered glutamine-associated metabolites [8]. In terms of potential mechanisms, visceral adiposity may promote progression from BO to OAC, via several metabolic and inflammatory pathways that are coincident with VAT [9]. Visceral obesity increases circulating metabolites and related hormones, including glucose, free fatty acids, triacylglycerol (TAG), ceramides, insulin, insulin growth factor (IGF), and a suite of inflammatory adipokines [[10], [11], [12], [13]]. This combined metabolic, hormonal, and inflammatory milieu may result in anti-apoptosis, mitogenic, pro-angiogenic, and immune suppressive processes driving tumorigenic capabilities.

It is important to appreciate that obesity is highly heterogeneous [14]. Therefore, the challenge is to define whether specific fatty acids, which have been shown to dictate obese phenotypes, differentially affect cancer progression. We and others have demonstrated that dietary fat composition can determine adipose tissue morphology and the associated immuno-metabolic phenotype. Feeding a high-fat diet (HFD) rich in saturated fatty acids (SFA) induced primed and activated adipose NLRP3 mediated IL-1β inflammation, with enhanced TNFα, and IL-6 expression, coupled with insulin resistance (IR). In contrast, feeding a monounsaturated fatty acid (MUFA) rich HFD induced a hyperplastic expansion of adipose, little or no adipose SVF-derived inflammation, and attenuated IR, despite equal obesity [15]. This observation has been verified in humans, wherein overfeeding a SFA-rich diet specifically expands the visceral adipose depot [16]. Hypertrophic visceral adipose results in a greater risk of metabolic disease and OAC [17,18]. Given that SFA and MUFA have differential effects on obese adipose phenotypes, it is important to better understand their specific roles in OAC progression.

Research into the role of SFA and MUFA in tumorigenesis is increasing. Cell proliferation pathways, such as ERK1/2-mTOR-NF-κB and PI3K/Akt, are modulated differently by SFAs and MUFAs, depending on the specific model and type of cancer [19]. These findings imply that different lipids can uniquely influence tumorigenesis. In the progression from BO to OAC, SFA palmitate increased the expression of CPT1A in both in vitro and in vivo models leading to enhanced cell proliferation [20]. Notably, CPT1A is a rate-limiting enzyme in fatty acid oxidation that has been associated with promoting cancer cell proliferation [21]. MUFA oleate decreased cell proliferation in oesophageal cancer cell lines OE19 and OE33 by enhancing AMPK phosphorylation. Moreover, oleate upregulated the tumour suppressor genes p53, p21, and p27 expression [22]. Overall, there is still much that is unknown about the role of individual fatty acids in the regulation of tumour growth and metastasis in BO and OAC.

Given obesity is associated with oesophageal cancer risk, and fatty acids dictate adipose tissue immune-metabolic properties and cell proliferation, this study addressed whether SFA would drive markers of cancer initiation/progression in obesity-linked BO and OAC while MUFA may not. We demonstrate that SFA significantly increased inflammation, disrupted metabolism, and increased mitochondrial dysfunction compared to MUFA. Furthermore, these alterations were more pronounced within the BO models versus an OAC disease stage. These results highlight the potential for dietary fatty acid manipulation for the reduction of obesity-linked BO progression to OAC.

## Materials and methods

### Cell culture

Cells representing the oesophageal transition from metaplasia to oesophageal adenocarcinoma were cultured to answer how different fatty acids affect progressive stages of oesophageal adenocarcinoma. QH (or CP-A) cell lines representing Barrett’s oesophagus were grown in Bronchial Epithelial Cell Growth Basal Medium (BEBM) (2 mM glutamine, 10% fetal bovine serum (FBS), 1% penicillin-streptomycin) supplemented with BEBM SingleQuots (2 mL bovine pituitary extract, 0.5 mL insulin, 0.5 mL hydrocortisone, 0.5 mL Gentamicin sulfate – Amphotericin (GA-1000), 0.5 mL retinoic acid, 0.5 mL transferrin, 0.5 mL triiodothyronine, 0.5 mL adrenaline, and 0.5 mL human epidermal growth factor (hEGF) per 500 mL media) (Lonza, Switzerland). OE33 cells, representing OAC, were grown in Roswell Park Memorial Institute (RPMI) medium (2 mM glutamine, 10% FBS, 1% penicillin-streptomycin) (Gibco, United States). QH cell lines were obtained from the American Type Culture Collection (ATCC) (LGC Standards, United Kingdom) and OE33 cells were collected from the European Collection of Cell Cultures (Salisbury, United Kingdom).

### Fatty acid treatments

Oleate (Merck, Germany) and palmitate (Sigma Aldrich, United States) were conjugated to fatty acid-free bovine serum albumin (BSA) (Sigma Aldrich, United States), at a molar ratio of fatty acid (FA): BSA of 2:1. QH and OE33 cells were treated with vehicle control BSA, 250 μM palmitate or oleate for 48 hours. Cell culture supernatants were stored at -80°C. Cells were used for protein extraction, Seahorse analyzer, mass-spectrometry proteomics, or flow cytometry.

### Multiplex ELISA

Meso Scale Discovery (MSD) U-PLEX multiplex assay was used to assess the inflammatory profile from cell culture supernatants following FA treatments (Meso Scale Diagnostics, United States). A U-PLEX Biomarker Group 1 (Human) assay was customised to profile *IL-1β, IL-6, IL-8,* and *TNFα*. Each biotinylated antibody was coupled with a unique linker. Each U-PLEX Linker-coupled antibody solution is added to a single tube to make a multiplex coating solution. Each well in a 96-well plate received 50 μL of the antibody solution and the plate was sealed. The plate was stored overnight (O/N) at 4°C. The following day, the calibrator standards 1, 3, and 9 were prepared. Each calibrator vial was reconstituted with Diluent 43 to a concentration of 5x and incubated for 30 minutes at room temperature. Each calibrator was diluted to 1x and used at Standard 1. The subsequent 6 dilutions were made in diluent 43 at 4-fold serial dilutions. Calibrator standards and cell culture supernatants were added to each well and the plate was incubated O/N at 4°C. On day 3, the plate was washed 3x with PBS-Tween. The detection antibody solution was made by combining each detection antibody into one tube and down to 1 x from 100x in diluent 3. Detection antibody solution was added to each well and incubated at room temperature for 1 hour while shaking. The plate was washed 3x with 0.5% PBS-Tween. MSD GOLD Read Buffer B was added to each well. The assay was run on a MESO QuickPlex SQ120, and all analyte concentrations were calculated using Discovery Workbench software (version 4.0). Measurements were normalised to cellular lysate protein content quantified using a BCA assay (Pierce, Thermo Scientific, United States).

### Real-time ATP rate assay

QH and OE33 cells were seeded at 7,000 or 10,000 respectively in a 24-well cell culture XF microplate (Agilent Technologies, United States). After 24 hours, cells were untreated, treated with BSA vehicle control, 250 μM palmitate, or oleate. 48 hours following treatment, cells were rinsed with Seahorse XF DMEM Medium (pH 7.4, 10 mM XF glucose, 1 mM of XF pyruvate, 2 mM of XF glutamine) (Agilent Technologies, United States). Cells were incubated in Seahorse XF DMEM Medium for 1 hour at 37°C in a non-CO_2_ incubator. The sensor cartridge was loaded in port A with oligomycin (1.8 μM, Sigma Aldrich, United States) and port B with antimycin A (2 μM, Sigma Aldrich, United States). Seahorse XF Real-Time ATP Rate Assay was completed with Seahorse XFe24 Analyzer (Agilent Technologies, United States). Three basal oxygen consumption rate (OCR) and extracellular acidification rate (ECAR) were measured before oligomycin injection. This allowed for an inhibition of mitochondrial ATP synthesis thus quantification of mitoATP Production Rate. Three additional OCR and EACR measurements were taken before injection with antimycin A. Mitochondrial respiration inhibition by antimycin A allowed for the calculation of the glycoATP Production Rate. Measurements were normalised to cell number by crystal violet assay.

### Mito stress assay

QH and OE33 cells were seeded at 10,000 or 15,000 respectively in a 24-well cell culture XF microplate (Agilent Technologies, United States). The following day, cells were either untreated, treated with BSA vehicle control, 250 μM palmitate, or oleate. After 24-hour treatment, cells were rinsed with Seahorse XF DMEM Medium (pH 7.4, 10 mM XF glucose, 1 mM of XF pyruvate, 2 mM of XF glutamine) (Agilent Technologies, United States). Cells were incubated in Seahorse XF DMEM Medium for 1 hour at 37°C in a non-CO2 incubator. The sensor cartridge was loaded in port A with oligomycin (1.8 μM, Sigma Aldrich, United States), port B with carbonyl cyanide-p-trifluoromethoxyphenylhydrazone (FCCP) (2 μM, Sigma Aldrich, United States) and port C with antimycin-A (2 μM, Sigma Aldrich, United States). Seahorse XF Real-Time Mito Stress Assay was completed with Seahorse XFe24 Analyzer (Agilent Technologies, United States). Three basal OCR and ECAR were measured before oligomycin injection. This allowed for an inhibition of mitochondrial ATP synthesis thus quantification of cellular ATP production. Three additional OCR and EACR measurements were taken before injection with FCCP. FCCP uncouples the proton gradient and disrupts the mitochondrial membrane potential. The OCR stimulated by FCCP is used to measure spare respiratory capacity. Mitochondrial respiration inhibition by antimycin A allowed for the calculation of nonmitochondrial respiration. Measurements were normalised to cell number by crystal violet assay.

### Crystal violet assay

Cell media was removed carefully from cells and stored at -20°C. Fifty μL 1% glutaraldehyde (Gibco, United States) (in PBS) was added per well and incubated for 20 minutes at room temperature. The glutaraldehyde was removed, and cells were gently washed 1x with PBS. Fifty μL 0.1% crystal violet (Sigma Aldrich, United States) (in ddH2O) was added to each well for 30 minutes at room temperature, washed 1x with H_2_O and dried overnight. The following day, 50 μL of 1% triton-X (in PBS) was added to each well and agitated for 15 minutes at room temperature. Each corresponding well solution was then transferred into a new 96 well plate and absorbance read at 595 nm on a CLARIOstar plate reader (BMG Labtech, Germany).

### Mass-spectrometry proteomics

After 48-hour fatty acid treatments, cells were trypsinised and snap-frozen at -80°C. Frozen cell pellets were resuspended in 8 M Urea/50 mM Tris HCl and sonicated. Samples were adjusted for 200 μg total protein, then reduced by the addition of 8 mM dithiothreitol and mixed at 30°C for 30 mins at 1000 rpm. Samples were carboxylated by the addition of 20 mM iodoacetamide and mixed at 30°C for 30 mins at 1000 rpm in the dark. Samples were diluted to below 1 M urea with 50 mM Tris HCl. Samples were digested overnight at 37°C in trypsin (Promega, Madison, Wisconsin, United States) (0.5 μg enzyme per μg protein). Digestion was terminated by the addition of formic acid to a final concentration of one percent. Samples were cleaned through HyperSep Spin Tips. SpinTips were wet with elution buffer (60% acetonitrile (ACN) in 0.1% trifluoroacetic acid (TFA)) and centrifuged at 2500 rpm for 2 minutes. Tips were equilibrated with 0.1% TFA in liquid chromatography-mass spectrometry (LC-MS) grade H_2_O and centrifuged at 2500 rpm for 2 mins. Proteins were bound in SpinTips and centrifuged at 2500 rpm for 2 minutes. Samples were eluted with 60% ACN in 0.1% TFA and centrifuged for 2 mins. Samples were evaporated in an eppendorf concentrator at 45°C. All reagents were from Sigma Aldrich, United States.

*LC-MS/MS Method (Bruker timTof Pro (Bruker, Billerica, United States) /Evosep One (Evosep, Denmark))* Tryptic samples were resuspended in 0.1% formic acid in ACN and loaded onto Evotip Pure EV-2001 MAT C:18 tips (Evosep, Denmark). Peptides were separated on an Evosep EV1137 Performance Column – 15 cm x 150 μm, 1.5 μm. The Evosep tips were placed in position on the Evosep One, in a 96-tip box. The autosampler is configured to pick up each tip, elute, and separate the peptides using a set chromatography method (15 samples a day) [23]. The mass spectrometer was operated in positive ion mode with a capillary voltage of 1150 V, dry gas flow of 3 l/min, and a dry temperature of 180°C. All data was acquired with the instrument operating in trapped ion mobility spectrometry (TIMS) mode. Trapped ions were selected for ms/ms using parallel accumulation serial fragmentation (PASEF). A scan range of (100-1700 m/z) was performed at a rate of 5 PASEF MS/MS frames to 1 MS scan with a cycle time of 1.03s [24].

*MaxQuant Analysis* The raw data was searched against the Homo sapiens subset of the UniProt Swissprot database (reviewed) using the search engine MaxQuant (release 2.0.3.0) using specific parameters for TIMS data-dependent acquisition (DDA). Each peptide used for protein identification met specific MaxQuant parameters, i.e., only peptide scores that corresponded to a false discovery rate (FDR) of 0.01 were accepted from the MaxQuant database search. The normalised protein intensity of each identified protein was used for the generation of label-free quantitation (LFQ) [25]. The Perseus computational platform (version 1.6.15.0) was used to process MaxQuant results [26]. Data was log-transformed. Analysis of Variation (ANOVA) and *T*-test comparisons were carried out between cell treatments. For visualisation of the data using heat maps, missing values were imputed with values from a normal distribution and the dataset was normalised by z-score. Clustering was completed in Cluster 3.0 and visualised in TreeView [27]. The mass spectrometry proteomics data have been deposited to the ProteomeXchange Consortium via the PRIDE [28] partner repository with the dataset identifier PXD055313.

### Bioinformatic pathway analysis and protein-protein network analysis

Bioinformatic analysis was performed to analyse differentiable expressed cellular proteins. Briefly, t-test differences from the proteomic analysis were uploaded into the Reactome pathway knowledgebase [29]. The Reactome knowledgebase provides predictive biological processes and functional relations. Protein-protein network analysis was performed by STRING [30] in Cytoscape (version 3.10.1).

### Flow cytometry for mitochondrial profiling

Cells were seeded at 80,000 cells per well in 24 well plates. The following day, cells were treated with either BSA vehicle control, palmitate, or oleate for 48 hours. After treatment, cells were stained for flow cytometry. For mitochondrial profiling and lipid accumulation, cells were stained with Mitosox Red mitochondrial superoxide indicator on PE [5 uM] (Fisher Scientific, Ireland), Mitotracker Orange CMTMRos on PE-Cy5.5 (Fisher Scientific, Ireland) [100 nM], HCS LipidTOX Green neutral lipid stain [600x] on FITC (Fisher Scientific, Ireland) and 4,6-Diamidino-2-Phenylin, dihydrochloride (DAPI) on Pacific Blue [1 ug/mL] (Fisher Scientific, Ireland). Flow cytometry samples were acquired using Amnis® CellStream® (Luminex, United States) and analysis was conducted on single cell, DAPI+ cells with CellStream® analysis software.

### Western blotting

Protein separation was performed in a 10% polyacrylamide gel using a BIO-RAD system. After electrophoresis, gels were transferred to 0.45 μM PVDF membranes (Sigma Aldrich, United States) using a wet-type blotting system. Membranes were blocked in 5% skim milk in TBS-T. After membranes were blocked, the membrane was probed with primary antibody rabbit polyclonal anti-Akt (Cell Signaling, United States) overnight at 4°C. After washing 5 times with TBS-T, the membrane was incubated with secondary antibody (anti-rabbit HRP-conjugated from Cell Signaling, United States) for 1 h at room temperature. After washing with TBS-T, the membranes were incubated with Immobilon Western Chemiluminescent HRP Substrate (Sigma Aldrich, United States) and visualised on Vilbur Fusion Fx7. Protein band intensity was quantified using ImageJ [31].

### Statistical analysis

Data are expressed as mean ± standard error (S.E.M.). Statistical analysis for cytokine secretion, Seahorse Analyzser, flow cytometry and protein expression by western blot was completed using GraphPad Prism 10.0 (GraphPad Prism, United States). For between-group comparisons, a one-way ANOVA followed by a post-hoc Bonferroni test was performed in order to test for differences in means between all 3 groups. For OCR and EACR values throughout the Mito Stress Assay, a two-way repeated measures ANOVA was completed, followed by a post-hoc Bonferroni test. For proteome results, statistical analysis was performed in Perseus (version 1.6.15.0). LFQ values were log-transformed. ANOVA and *T*-test comparisons were carried out between cell treatments. Differences for heat map visualisation were obtained after missing values were imputed from a normal distribution and the dataset was normalised by z-score. A p-value <0.05 was considered statistically significant.

## Results

### Saturated fatty acid increases inflammation in BO to a greater extent than OAC, compared to monounsaturated fatty acid

**Fig. 1:**
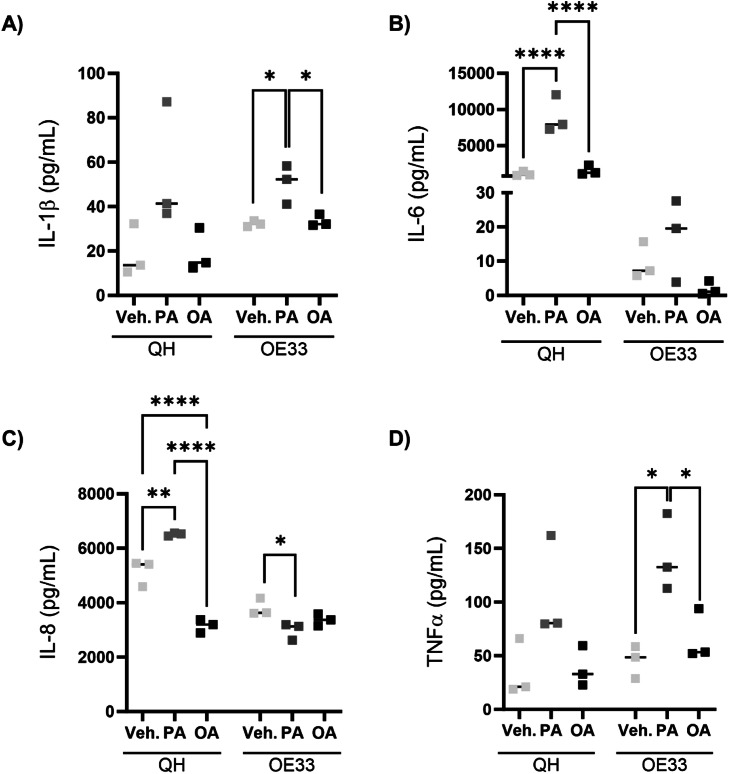
Palmitate increases inflammation in both metaplasia and oesophageal adenocarcinoma stages. Pro-inflammatory cytokine secretion of (A) IL-1β, (B) IL-6, (C) IL-8 and (D) TNFα from QH and OE33 treated with 250 μM palmitate (PA) or oleate (OA) for 48 hours. Fatty acids (FA) were conjugated to bovine serum albumin (BSA) at a FA: BSA molar ratio of 2:1. BSA, bovine serum albumin; FA, fatty acid; IL-1β, interleukin 1β; IL-6, interleukin 6; IL-8, interleukin 8; TNFα, tumour necrosis factor α; Veh., vehicle (BSA). *p<0.05, **p<0.01, ****p<0.0001.

Fatty acids can induce distinct inflammatory profiles. However, their differential impact between the different stages of BO versus OAC is unknown. We investigated the impact of the SFA palmitate (PA) versus the MUFA oleate (OA) treatment in QH cells, representing BO and OE33 cells, which represent OAC. In the OE33 OAC cell line, PA induced a pro-inflammatory cytokine response, characterised by significantly increased IL-1β while QH IL-1β levels were not significantly changed (Fig. 1A). PA treatment significantly increased IL-6 secretion in QH cells but not OE33 cells, compared to vehicle control and OA treatments (Fig. 1B). Interestingly, PA induces exponentially more IL-6 levels compared to OE33 cells. In QH cells, PA increased IL-8 secretion while OA reduced it, compared to control. Interestingly, PA reduced IL-8 secretion from OE33 cells (Fig. 1C). Moreover, IL-8 levels were higher in QH cells compared to OE33 following PA incubation. PA treatment also increased QH TNFα secretion, albeit not significantly. In contrast, OA fatty acid treatment did not induce a pro-inflammatory response having similar concentrations to vehicle. Alternatively, in OE33 cells, PA increased TNFα secretion (Fig. 1D). Again, OA did not modulate an inflammatory response in OE33 cells. These results suggest that in oesophageal cancer cells saturated fatty acids, specifically PA, may be particularly deleterious with respect to the inflammatory status. The increase of IL-6 and IL-8 in QH and IL-1β and TNFα in OE33 indicates the cytokine signature may be dependent upon the progressive stages of BO versus OAC.

### Saturated and monounsaturated fatty acids induce differential cellular proteome expression

**Fig. 2:**
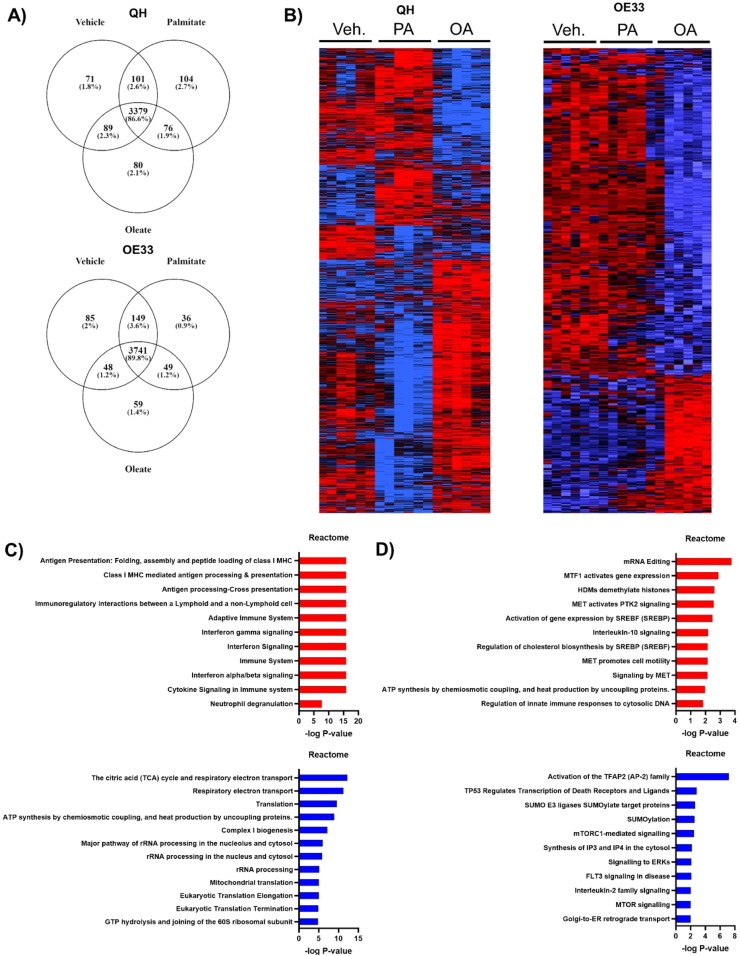
Palmitate versus oleate alters predicted cellular pathways in metaplasia and oesophageal adenocarcinoma states. (A) Venn diagram showing the number and overlap of proteins identified for QH and OE33 following fatty acid treatments. (B) QH and OE33 proteomic signature heat maps. Red and blue bars indicate proteins significantly up- or down-regulated respectively p<0.05. Reactome pathways in (C) QH and (D) OE33 cells upregulated in palmitate treatments compared to oleate in red or upregulated in oleate treatments compared to palmitate in blue. OA, oleate; PA, palmitate; Veh., vehicle (bovine serum albumin (BSA)).

Based on the differential effect of fatty acids on the inflammatory profile of BO versus OAC cell models, we performed an unbiased mass-spectrometry (MS)-based proteomics approach to get a deeper understanding of how the different fatty acids affect markers of cancer progression (Fig. S1). Proteomics results were validated by western blots (Fig. S2). In QH cells, a total of 3640 proteins were identified with vehicle treatment, 3660 proteins identified with PA treatment, and 3624 proteins identified with OA treatment. Remarkably, we show that 71 proteins were exclusive to the vehicle, 104 were exclusive to PA and 80 were exclusive to OA. Overall, we see that 3455 proteins overlapped between PA and OA treatments (Fig. 2A). There were more proteins identified in OE33 cells with 4,023 in vehicle-treated cells, with PA treatment, a total of 3975 and only 3897 proteins identified within OA treatment. There were 3790 total proteins that overlapped between OA and PA treatments (Fig. 2A). In QH cells, vehicle and PA treatments produced 182 differentially expressed proteins with 96 increased in vehicle and 86 increased in PA. Vehicle treatments compared to OA produced 221 differentially expressed proteins with 110 proteins increased in the vehicle and 111 increased in OA. There were a total of 821 differentially expressed between PA and OA treatments, with 332 increased in abundance when the cells were treated with PA and 489 proteins were decreased with this treatment when compared to cells treated with OA. Similarly, differences in the proteome of OE33 cells were present when treated with OA or PA albeit fewer proteins were differentially expressed in these cells a total of 148. In particular, there were 108 increased in expression and 40 proteins decreased. A total of 435 proteins differentially expressed between the vehicle control and OA. We saw an increase of 299 proteins expressed in BSA and 136 increased in OA. Interestingly, there was no significant difference in proteins between vehicle and PA (Fig. 2B) (Fig. S3A&B). Altogether, these results demonstrated that we saw different protein expression dependent on cell type and treatment.

To gain a better insight into our dataset, we focused on differentially regulated proteins. In QH cells, proteins that were significantly increased in PA compared to OA include the Ras related in brain (RAB) oncogene family (RAB5A, RAB18, RAB2B) and lipid metabolism (acyl-CoA synthetase long chain family member (ACSL) 4, stearoyl-CoA desaturase (SCD)). In OE33 cells, PA treatment compared to OA increased markers of lipid metabolism (acyl-CoA synthetase (ACS) members ACSS2, ACSF3). Furthermore, mitochondrial proteins, involved with mitochondrial energy metabolism were increased by PA, in comparison to OA treatment, in both QH (Mitochondrially Encoded Cytochrome C Oxidase I (MT-CO1), acetyl-CoA acetyltransferase (ACAT) 1) and OE33 (ATP5J2, NADH:ubiquinone oxidoreductase subunit (NDUF) S2/B6) cells. Thus, the differentially expressed proteins indicated that the treatments with the two fatty acids cause specific functional changes in each cell type. To confirm this, we performed an analysis to identify enriched functional molecular pathways. Using the Reactome pathway database, we confirmed that biological processes were significantly differentially expressed between PA and OA treatments in QH cells. PA treatment upregulated several immune pathways including antigen processing/presentation, immune system and interferon signalling, and cytokine signalling in the immune system compared to OA (Fig. 2C). These pathways were not affected by PA treatment, compared to vehicle however, there was another cross-presentation pathway upregulated that involved soluble exogenous antigens (Fig. S4A). OA treatment increased pathways relating to mitochondrial function and lipid metabolism in QH cells, compared to PA treatment. Within mitochondrial function, these processes include ATP metabolic processes (i.e. cytochrome c oxidase (COX) members COX4I1, COX6A1, COX7C), mitochondrial protein synthesis (i.e. mitochondrial ribosomal protein (MRP) MRPL and MRPS family), and respiratory chain (i.e. NDUFA9, NDUFS8) amongst others (Fig. 2C). This indicates that mitochondrial function was decreased by PA treatment. In contrast, OA treatment also increased respiratory electron transport and ATP synthesis, compared to vehicle control (Fig. S4B). With OE33 cells, PA increased ATP synthesis pathways, compared to OA. Alternatively, OA displayed an increase in signalling to ERKs, IL-2 family signalling, and mTOR signalling (Fig. 2D). OA treatment also increased the amino acids that regulate the mTORC1 pathway, compared to vehicle (Fig. S4C).

Gene ontology (GO) pathways were also utilised to identify enriched biological processes. Within GO biological terms following PA, relative to OA, we observed an increase in lipid metabolic processes, lipid oxidation, fatty acid metabolic processes, and fatty acid β-oxidation (Fig. S5A). In the case of OE33 cells, there were fewer GO biological processes different between the two fatty acid treatments. Specific to mitochondrial activity, there was an increase in the oxygen-reduction process in PA compared to OA. Additionally, there was an enrichment in cholesterol metabolism with an increase in cholesterol biosynthetic process and metabolic process (Fig. S5B).

**Fig. 3:**
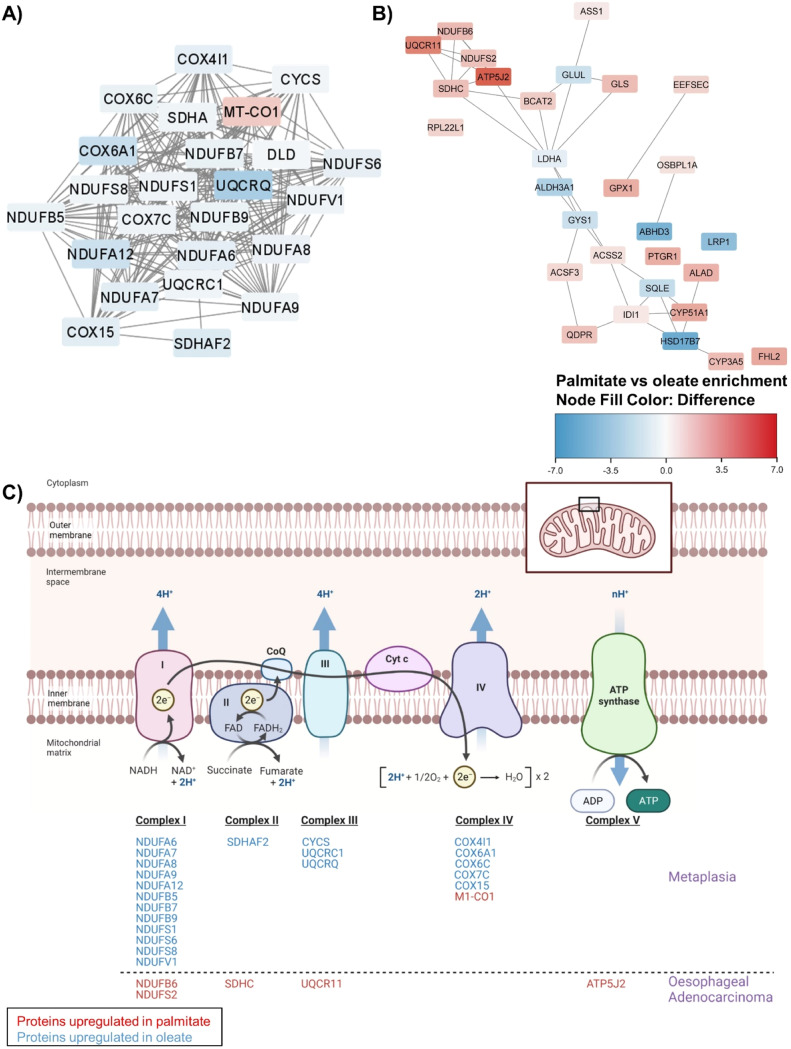
Palmitate versus oleate impacts on metabolic protein-protein interaction networks in OAC disease models. Protein-protein metabolic networks in (A) QH and (B) OE33 cells. (C) Proteome changes are mapped to different elements of the electron transport chain in the metaplasia model (QH) and the oesophageal adenocarcinoma model (OE33). Red indicates proteins significantly enriched with palmitate and blue indicates proteins significantly enriched with oleate p<0.05. This Fig. was created using Biorender.com.

To gain insight on the implication that the changes of protein expression might have in the molecular network we used STRING to reconstruct protein networks affected by the treatment in each cell. Protein-protein networks represent protein connections within the cells. Remarkably inferred protein-protein interactions show quite a difference in mitochondrial complex proteins between PA and OA in both QH and OE33 cells (Fig. 3A&B). These interactions highlight following OA treatment in QH cells, there is an increase in NDUF family expression compared to PA which represents the regulation of oxidative phosphorylation (Fig. 3A). This indicates that PA treatment reduces the electron transport chain, compared to OA treatments. Furthermore, these interactions highlight the differential effects that PA versus OA have in QH cells specifically with SCD activity, indicative of fatty acid synthesis, increasing with PA and having a central role in the lipid metabolic process. Within OE33, more metabolic differences are seen within mitochondrial enzymatic and complex proteins increasing in PA compared to OA (ATP5J2, NDUFB6, NDUFS2) (Fig. 3B). Combining the results from the Reactome pathways, GO Biological processes, and STRING protein-protein networks, it was evident that the different fatty acids greatly impact mitochondrial activity. Specifically, these proteome changes can be mapped to different complexes along the electron transport chain (Fig. 3C). The proteome changes in PA versus OA treatments show that PA reduces oxidative phosphorylation while increasing lipid metabolism in comparison to OA. These alterations are indicative of markers of cancer progression and support that different fatty acids may have different effects on cancer progression within both BO and OAC cell models however, more alterations are occurring earlier in the disease progression.

### Palmitate causes mitochondrial dysfunction in QH cells while making OE33 cells more glycolytic

**Fig. 4:**
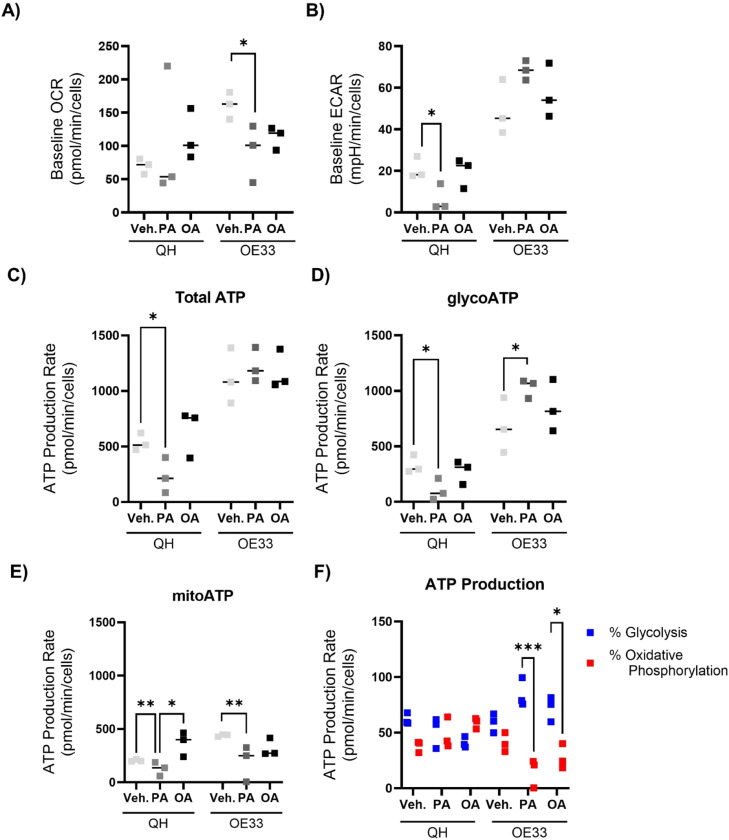
ATP production rates differ between fatty acid treatments. Baseline OCR (A), baseline ECAR (B), total (C), glycolytic (D) and mitochondrial ATP (E) production rates from QH cells and OE33 following fatty acid treatments. (F) Percentage of glycolysis and oxidative phosphorylation in both QH and OE33 cells. These cells are treated with 250 μM palmitate or oleate for 48 hours. ATP, adenosine triphosphate; ECAR, extracellular acidification rate; glycoATP, glycolytic ATP; mitoATP, mitochondrial ATP; OA, oleate; OCR, oxygen consumption rate; PA, palmitate; Veh., vehicle. *p<0.05, **p<0.01, ***p<0.001.

Next, we validated the proteomics analysis that indicated significantly different effects of fatty acids on mitochondrial metabolism, by determining cellular bioenergetics and mitochondrial activity in real-time by Seahorse Analyzer. In QH cells, after 48 hours, neither treatment regulates the baseline OCR, which is a marker of normal mitochondrial cellular function. In OE33 cells, both PA and OA reduced the baseline OCR rate (Fig. 4A). In QH cells, PA treatments significantly reduced baseline ECAR, compared to control. ECAR is a measurement of glycolytic flux in cells. There was no difference in ECAR between OA and vehicle. Additionally, there was no difference in the baseline ECAR between treatments in OE33 cells (Fig. 4B). Overall, OE33 cells were more metabolically active than the QH cells indicated by their baseline OCR and ECAR. PA treatment significantly reduced total ATP production, due to a reduction in both glycolytic and mitochondrial ATP production. OA treatment increased the mitochondrial ATP production compared to PA. However, in comparison to PA, OA treatment did not modulate functional markers of mitochondrial and cellular energy metabolism in QH cells to the same extent. Whilst total ATP production in OE33 cells was not affected by the different fatty acids, the split between glycolytic and oxidative metabolism was quite different according to fatty acid treatments. PA greatly increased glycolytic ATP production concomitant with a reduction in mitochondrial ATP in OE33 cells, whereas OA treatment did not (Fig. 4C, D & E). In QH cells, the ratio between oxidative phosphorylation and glycolysis did not differ between treatments. Alternatively, both PA and OA treatments shift the cells towards a more glycolytic state in comparison to oxidative phosphorylation relative to control in OE33 cells (Fig. 4F). Altogether, these results showed that PA re-configured OE33 cell metabolism into a more glycolytic state, which concurs with their pro-inflammatory state. Following 24-hour treatment, neither QH nor OE33 cells displayed different baseline OCR or ECAR values (Figure S6A&B). During the mitochondrial stress test, mitochondrial respiration indicated by OCR trended to be lower while ECAR values were lower in PA-treated QH cells (Figure S6C). In OE33, OCR following uncoupling of mitochondrial oxidative phosphorylation, treatments with PA and OA had a lower mitochondrial response compared to the vehicle control represented by the OCR values. OE33 cells treated with PA had lower ECAR than OA or control (Figure S6D). Ultimately, PA was shown to have a greater effect on mitochondrial respiration in comparison to OA. These findings validate the proteomics results which suggested that PA treatment reduces the electron transport chain. Moreover, the mitochondria regulate multiple cellular processes altered in cancer cells including metabolism, apoptosis, and oxidative stress. This suggests that PA could drive cancer to a greater extent than OA.

### Saturated fat increases mitochondrial stress to a greater extent than monounsaturated fat

**Fig. 5:**
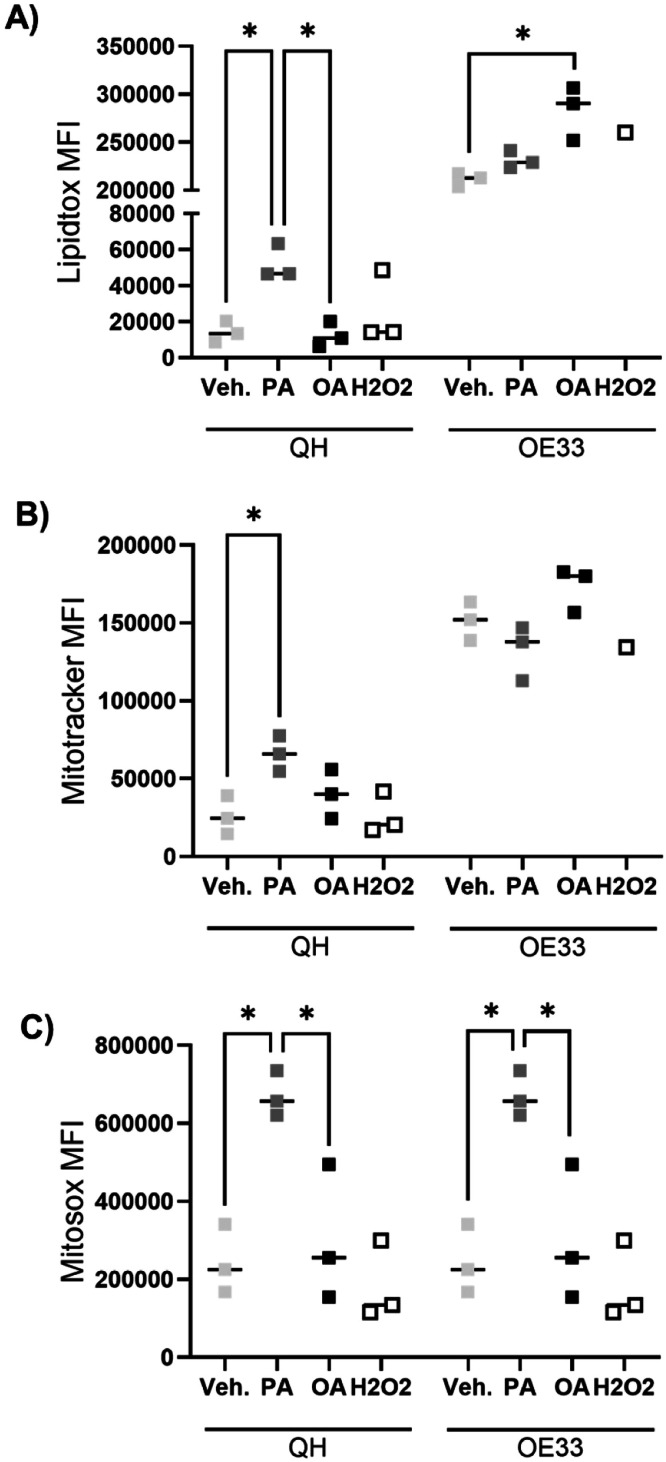
Fatty acid treatments change mitochondrial functionality. Flow cytometry analysis of (A) lipidtox, (B) mitotracker and (C) mitosox. QH and OE33 treated with 250 μM palmitate or oleate for 48 hours. H2O2; hydrogen peroxide; MFI, mean fluorescence intensity; OA, oleate; PA, palmitate; Veh., vehicle. *p<0.05.

Since PA increased markers of lipid metabolism and mitochondrial dysfunction to a greater extent than OA, in both BO and OAC models, we performed flow cytometry to understand the impact on lipid accumulation and mitochondrial stress. In QH cells, PA-treated cells displayed greater neutral lipid storage after 48 hours, with OA treatment similar to control levels. Interestingly in OE33 cells, OA displayed a higher lipid accumulation (Fig. 5A). When investigating mitochondrial properties, PA increased mitotracker stain, indicative of mitochondrial mass, following 48-hour treatment, compared to control. Remarkably, there was no significant difference in mitochondrial mass between the fatty acid treatments in OE33 (Fig. 5B). With both lipid accumulation and mitochondrial mass, QH and OE33 cells displayed different profiles following 48-hour fatty acid treatments. PA treatment increased reactive oxygen species (ROS) represented by mitosox stain, compared to the control in both QH and OE33 cells. In contrast, OA did not (Fig. 5C). The increase in ROS species in PA treatments corresponds to the increase in inflammatory cytokine secretion and a decrease in mitochondrial function seen in the inflammatory profile, proteome, and real-time metabolic profile.

## Discussion

Our study demonstrated that PA and OA had differential effects on the inflammatory profile, proteome and mitochondrial functionality at different stages of the BO to OAC transition. PA increased markers of cancer progression far greater than OA, in both pre-cancerous oesophageal cells (indicative of BO) and an established OAC cell model, albeit differently. PA treatment increased IL-6 and IL-8 secretion in the BO model, in comparison to OA. IL-6 has tumour-promoting behaviours which include downregulating the expression and activity of TP53 through increased TP53 protein degradation which results in enhanced evasion of growth suppression [32], cell death resistance [33,34], cancer cell self-renewal [35,36], and helping with tumour invasion and metastasis [[37], [38], [39]]. *In vitro*, malignant transformation of Barrett’s cell line that activated oncogenic Ras pathways and knockdown of the p53 pathway displayed increased IL-6 secretion [40]. Tumour burden is reduced in IL-6 knockouts while increasing IL-6 accelerated tumour formation [41]. An increase in IL-6 from PA, not OA, indicates that PA could drive cancer progression to a greater extent than OA. PA also increased IL-8 secretion in Barrett’s cells. RNA sequencing from OAC patients undergoing surgical resection showed an increase in IL-6 and IL-8 in the progression from BO to OAC [42]. IL-8 can modify tumour microenvironment composition [43]. Alternatively, in the OAC cell model PA reduced IL-8 secretion. Studies in oral epithelial expression of IL-8 demonstrated a decreased expression when the malignant transformation increases [44]. This could suggest that a decrease in IL-8 expression could still indicate a malignant transformation. Our data shows OE33 cells demonstrated an increase in IL-1β and TNFα with PA but not OA. Both IL-1β and TNFα have been shown to promote cancer progression through cell proliferation, migration, and metastasis [[45], [46], [47], [48]]. OE33 cells have a loss of function TP53 mutation C135Y [49]. The reduction in p53 activity results in loss of p53-mediated suppression of NF-κB leading to increased inflammatory response [50]. Furthermore, p53-knockout mice displayed elevated pro-inflammatory cytokines and increased NF-κB DNA binding activity in response to LPS stimulation [51]. Similar to LPS stimulation, PA activates TLR4 signalling to increase NF-κB translocation into the nucleus and enhances transcription of pro-inflammatory cytokines such as IL-1β and TNFα [13]. This suggests that the TP53 loss of function in OE33 is an important element which allows for an enhanced inflammatory response following PA treatment. This further demonstrates that PA can increase inflammation to a greater extent than OA in OAC cell models. The different inflammatory profiles between BO and OAC are not surprising due to their different expression of caspase-1 [52], whose activation is mediated by NLRP3, a key inflammasome that dictates inflammatory response to saturated fatty acids [53]. QH displayed expression of caspase-1 while OE33 cells did not, indicating the inflammatory mechanisms vary between different disease stages [52]. Additionally, it is important to highlight that inflammation displays dual roles in cancer being pro-tumorigenic in some scenarios and anti-tumorigenic in others [54]. Chronic inflammation, often displayed in obesity, can lead to immunosuppression and immune escape which leads to tumour progression. Our results show that this may be regulated by the class of fatty acid in the diet.

Mitochondrial activity was central to different responses witnessed and dependent on fatty acid treatments in both the metaplasia and adenocarcinoma stages. Pathway analysis showed an increase in mitochondria function in the metaplasia stage following OA treatment. These include ATP metabolic processes, mitochondrial protein synthesis, and the respiratory chain. Altered cellular bioenergetics, affecting metabolism, oxidative stress, and apoptosis, are fundamental to cancer initiation, development, and metastasis. Other studies support our data showing mitochondrial energy metabolism gene expression varies between the metaplasia to adenocarcinoma disease sequence with some genes decreasing (ATP12A, COX4I2) and others increasing (COX8C) [55]. Specifically, the ATP5B gene was significantly increased across this disease sequence shifting the cells to a more OXPHOS state. In the metaplasia state, PA downregulated multiple mitochondrial-related pathways including ATP synthesis, respiratory chain complex assembly, and mitochondrial translation. This was associated with reduced total, glycolytic, and mitochondrial ATP production with PA in comparison to control. OA was able to increase mitochondrial ATP production. PA has been shown to reduce mitochondrial function with a decrease in coupling efficiency, maximal respiration, and spare respiratory capacity while OA partially reverses these effects [56]. Mitochondria can generate ROS in the OXPHOS complexes and react with DNA, proteins, and lipids. ROS signalling needs to be tightly controlled since it can promote cancer cell growth but can also activate the cell death pathway [57]. It is well documented that higher ROS levels are in cancer cells compared to normal or pre-cancerous cells [58]. In the metaplasia stage, PA increased ROS species compared to OA. Oxidative stress is important to the transformation of BO to OAC. Patients with reflux-induced BO display oxidative stress which may contribute to the progression to OAC [59]. An increase in ROS can lead to an increase in pro-inflammatory IL-6 which was increased by PA. Overall, within the metaplasia state, PA disrupted mitochondrial function reducing ATP production and increasing ROS as well as mitochondrial content while OA did not have these effects. This suggests that PA increases markers of cancer progression in the metaplasia state through dysregulated mitochondrial activity while OA can maintain a less cancerous phenotype.

Our data also sheds light to the role of PA exposure in the regulation of lipid metabolism. PA displayed higher lipid metabolism expression with ACSL4 and SCD. ACSL4 is an Acyl-CoA synthetase (ACS) that converts fatty acid to fatty acyl-CoA esters. Dysregulation of ACSL4 has been reported in breast, colon, and gastric malignancies [[60], [61], [62]]. ACSL4 inhibition improves oesophageal tissue damage that is caused by chronic acid reflux [63] which drives the progression of Barrett’s oesophagus to oesophageal adenocarcinoma [18]. SCD converts SFA to MUFA which plays a role in cell growth, metabolic regulation, and signal transduction. In cancer, SCD-driven MUFA formation facilitates metabolic reprogramming through Akt, AMPK, and NF-κB regulation [64]. SCD-made MUFAs are used for phospholipids which are incorporated into dividing cancer cell membranes [64]. Inhibition of SCD suppresses oesophageal cancer progression displayed *in vitro* with reduced colony formation and *in vivo* with decreased tumour volume [65]. In line with these previous findings, our results in OE33 cells indicate that in the OAC model, PA increases markers of lipid metabolism with ACS family members ACSS2 and ACSF3. ACSS2 expression is increased in cancer cells that are under metabolic stress. Furthermore, ACSS2 expression was positively correlated with breast tumour stage and patient survival [66]. ACSF3 is seen to increase in malonate-related metabolism-related disease [67]. Malonate inhibits succinate dehydrogenase in the TCA cycle which then inhibits mitochondrial respiration. ACSF3 clears malonate through malonyl-CoA generation [68]. Altogether, the PA proteome signature indicates greater potential for oncogenic lipid metabolic expression compared to OA in the metaplastic stage, versus the adenocarcinoma stage.

An important observation of our study is that in the metaplasia stage, the proteome demonstrated distinct reconfiguration of the RAS-related oncogene family, specifically small GTPase superfamily members RAB5A, RAB18, and RAB2B, were increased with PA treatment, compared to OA. RAB family members regulate cell growth, survival, and apoptosis through signalling pathway activities [69]. RAB5A regulates intracellular vesicle transport resulting in cancer cell invasion and metastasis [70]. High RAB5A expression increased pancreatic cancer cell migration through increased filopodia formation [71]. Furthermore, activation of RAB5A promotes colorectal cancer progression [72]. Increased RAB18 expression was positively associated with poor prognosis in oesophagus squamous cell carcinoma following radiotherapy [73]. RAB2B levels are increased in colon adenocarcinoma [74]. Our data confirms the important role that RAB family of proteins could have in OAC progression. In particular within a metaplasia state, PA increases RAB family members' expression which is evident in cancer metastasis and treatment-resistant to a greater extent than OA.

In conclusion, we demonstrate that PA and OA elucidate very different effects on a precancerous versus cancerous stage in obesity-linked oesophageal disease. PA increases inflammation at both stages, albeit the cytokine signature is stage-dependent. Proteome change supports the difference in immune signalling and highlights lipid and mitochondrial metabolism perturbations largely driven by PA, not OA. Furthermore, the metabolic readout supports the proteome signature highlighting the difference in mitochondria functionality. There was more impact of PA at the pre-cancerous stage versus the cancerous stage. This highlights that different fatty acids ingested in the diet may have a greater impact early on in disease progression. These substantial differences between PA and OA highlight the importance of dietary interventions, both pre- and post-cancer development. Our study will inform future research into nutritional benefits to prevent cancer development. Furthermore, this highlights the importance of a nutrition component within oncology treatments which are focused on drug therapeutics. Precision nutrition paired with precision oncology research will allow us to fully elucidate the efficacy of dietary interventions on obese individuals who have a larger propensity to develop cancer.

## CRediT authorship contribution statement

**Kathleen A.J. Mitchelson:** Conceptualization, Data curation, Formal analysis, Investigation, Methodology, Validation, Visualization, Writing – original draft, Writing – review & editing. **Fiona O’Connell:** Data curation, Investigation, Methodology, Validation, Writing – review & editing. **Kieran Wynne:** Data curation, Investigation, Methodology, Writing – review & editing. **David Matallanas:** Data curation, Supervision, Writing – review & editing. **Jacintha O’Sullivan:** Conceptualization, Supervision, Writing – review & editing. **Helen M. Roche:** Conceptualization, Funding acquisition, Project administration, Resources, Supervision, Writing – original draft, Writing – review & editing.

## Declaration of competing interest

The authors declare that they have no known competing financial interests or personal relationships that could have appeared to influence the work reported in this paper.

## Appendix Supplementary materials

Image, application 1
