# Supplementary material for: Saturated fat exacerbates mitochondrial dysfunction through remodelling of ATP production and inflammation in Barrett’s oesophagus compared to monounsaturated fat, particularly in contrast to oesophageal adenocarcinoma
Source: Neoplasia. 2025 May 16;66:101173. doi: 10.1016/j.neo.2025.101173 (PMC12146558; doi:10.1016/j.neo.2025.101173)

Supplemental Information

Supplemental Figure 1 Mass Spectrometry Experimental Methodology. Graphical representation of the mass spectrometry purification protocol and subsequent analysis for QH and OE33 cells following fatty acid treatments. This figure was created using Biorender.com.

Supplemental Figure 2 Proteomics Validation by Western. Western blots and corresponding LFQ values of differentially expressed Akt between vehicle (Veh.), palmitate (PA) and oleate (OA) treatments in QH and OE33 cells.

Supplemental Figure 3 Volcano plots of differentially expressed proteins between vehicle, palmitate and oleate treatments (A) QH and (B) OE33 cells. OA, oleate; PA, palmitate; Veh., vehicle.

Supplemental Figure 4 Reactome pathways for QH cells (A) upregulated in vehicle versus palmitate (black) and upregulated in palmitate versus vehicle (red) and (B) upregulated in vehicle versus oleate (black) and upregulated in oleate versus vehicle (blue). In OE33 cells, Reactome pathways are upregulated in vehicle versus oleate in black and upregulated in oleate versus vehicle in blue (C). Red indicates pathways upregulated with palmitate, blue indicates pathways upregulated with oleate and black indicates pathways upregulated with vehicle.

Supplemental Figure 5 GO Biological Processes enriched in palmitate compared to oleate treatments in (A) QH and (B) OE33 cells.

Supplemental Figure 6 Mito Stress Assay. Baseline (A) OCR and (B) ECAR for QH and OE33 cells following fatty acid treatment. (C) OCR and ECAR Mito stress time course from QH post fatty acid treatment (D) OCR and ECAR Mito stress time course from OE33 post fatty acid treatment. These cells were treated with 250 µM palmitate or oleate for 24 hours. ECAR, extracellular acidification rate; OA, oleate; OCR, oxygen consumption rate; PA, palmitate; Veh., vehicle

Supplemental Figure 1

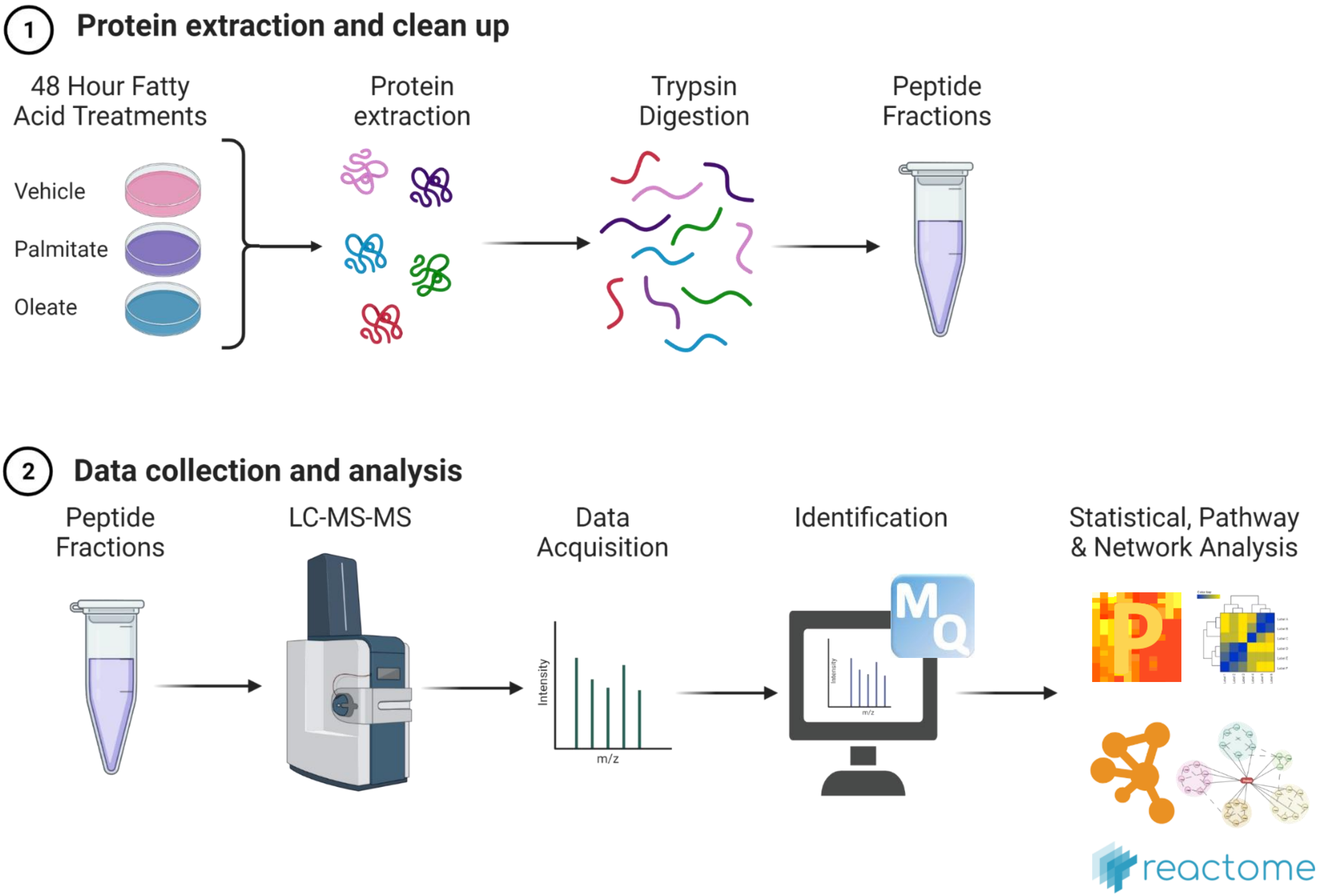

Supplemental Figure 2

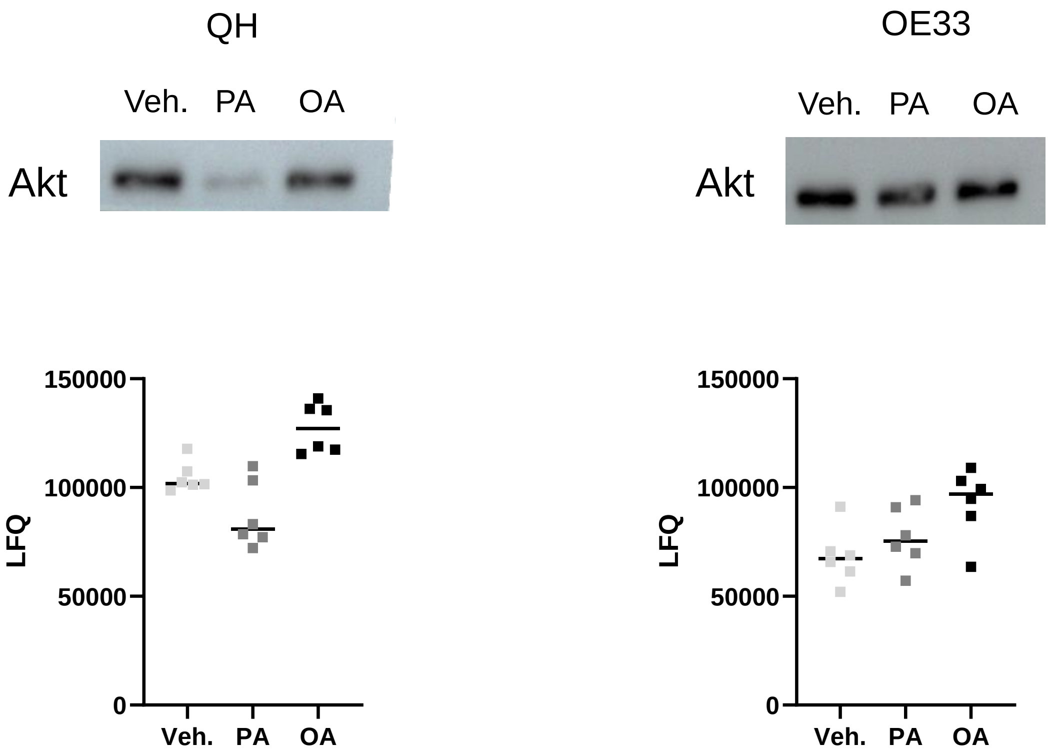

Supplemental Figure 3

A)

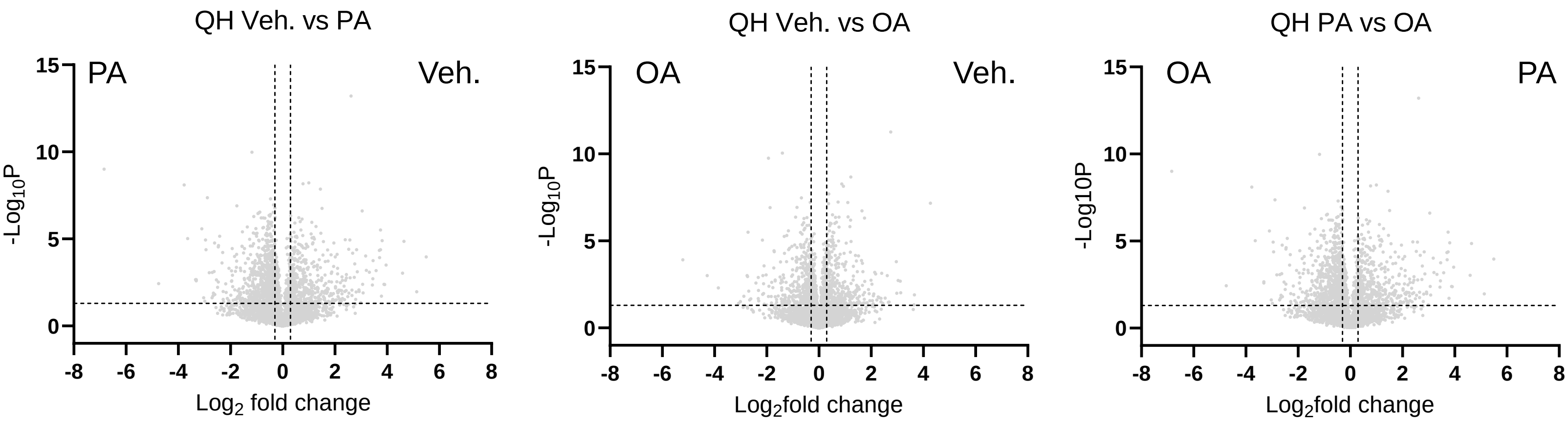

B)

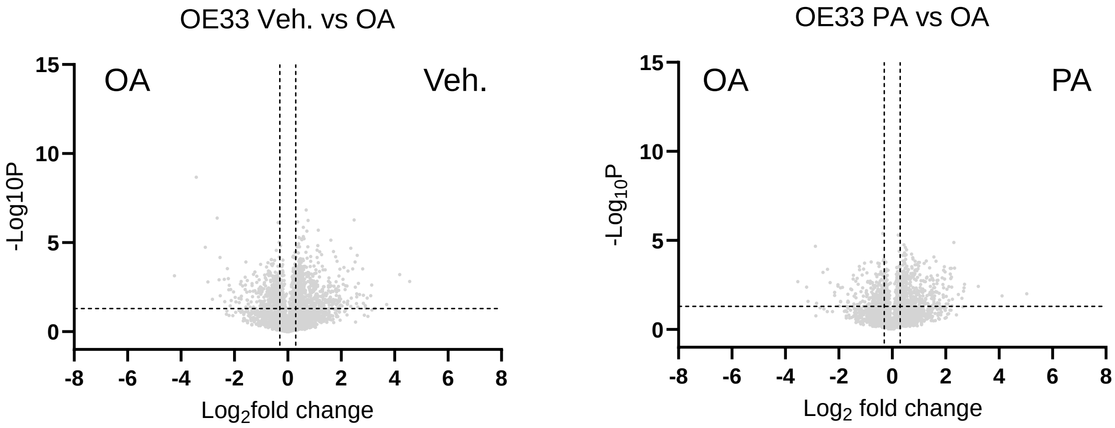

Supplemental Figure 4

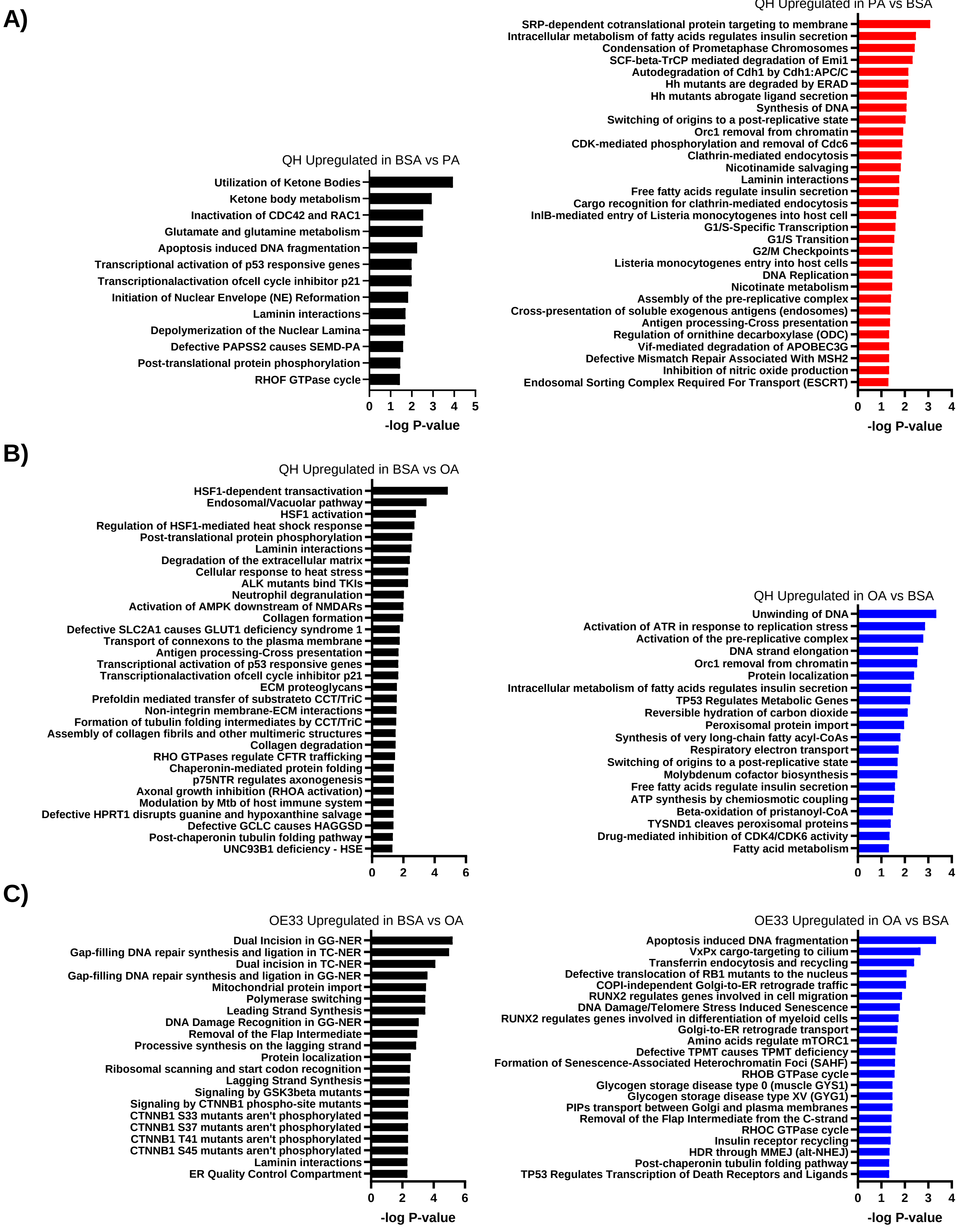

Supplemental Figure 5

A)

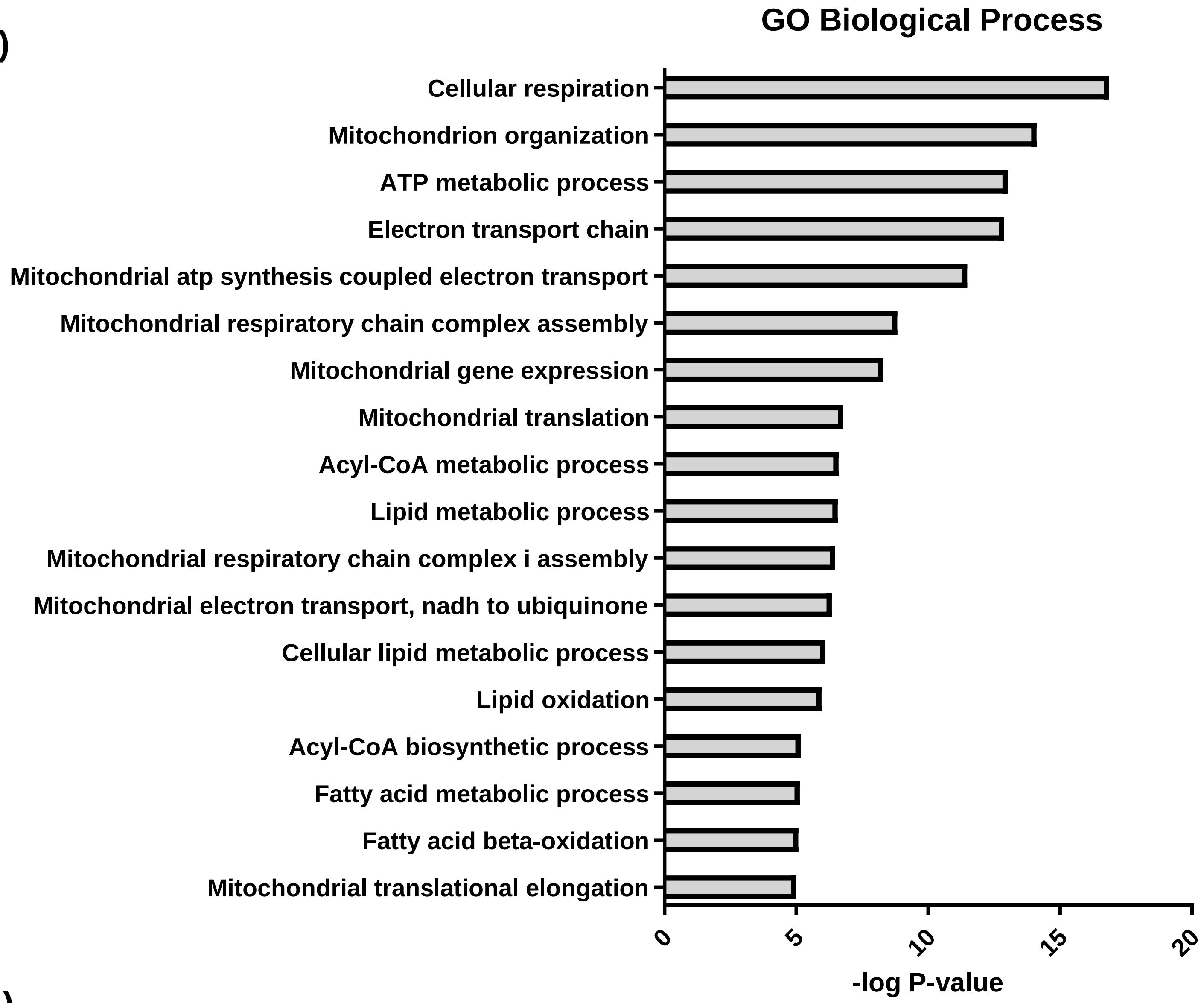

B)

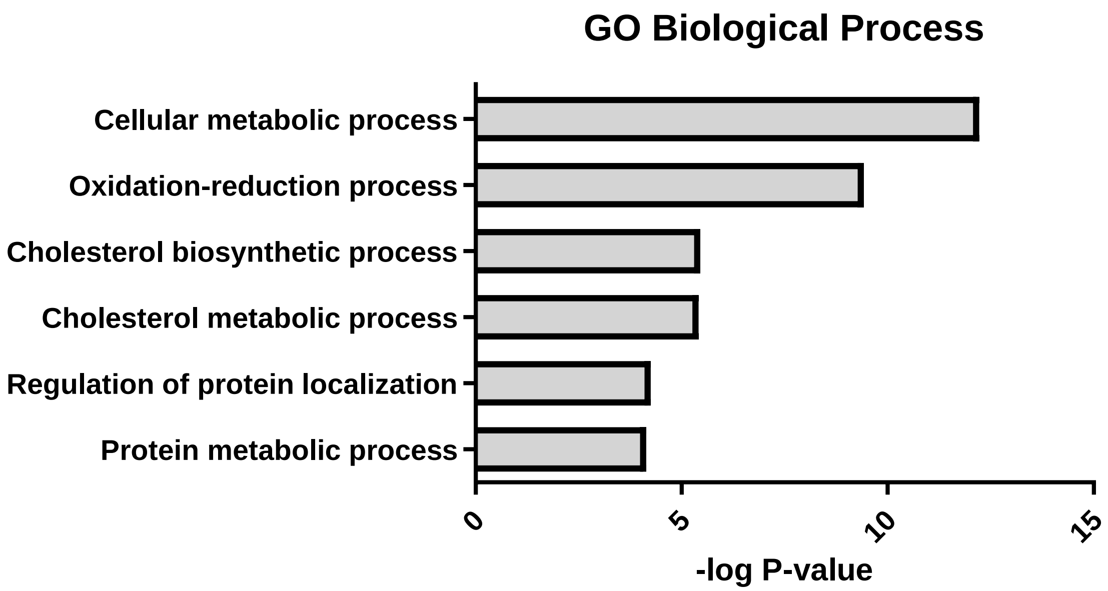

Supplemental Figure 6

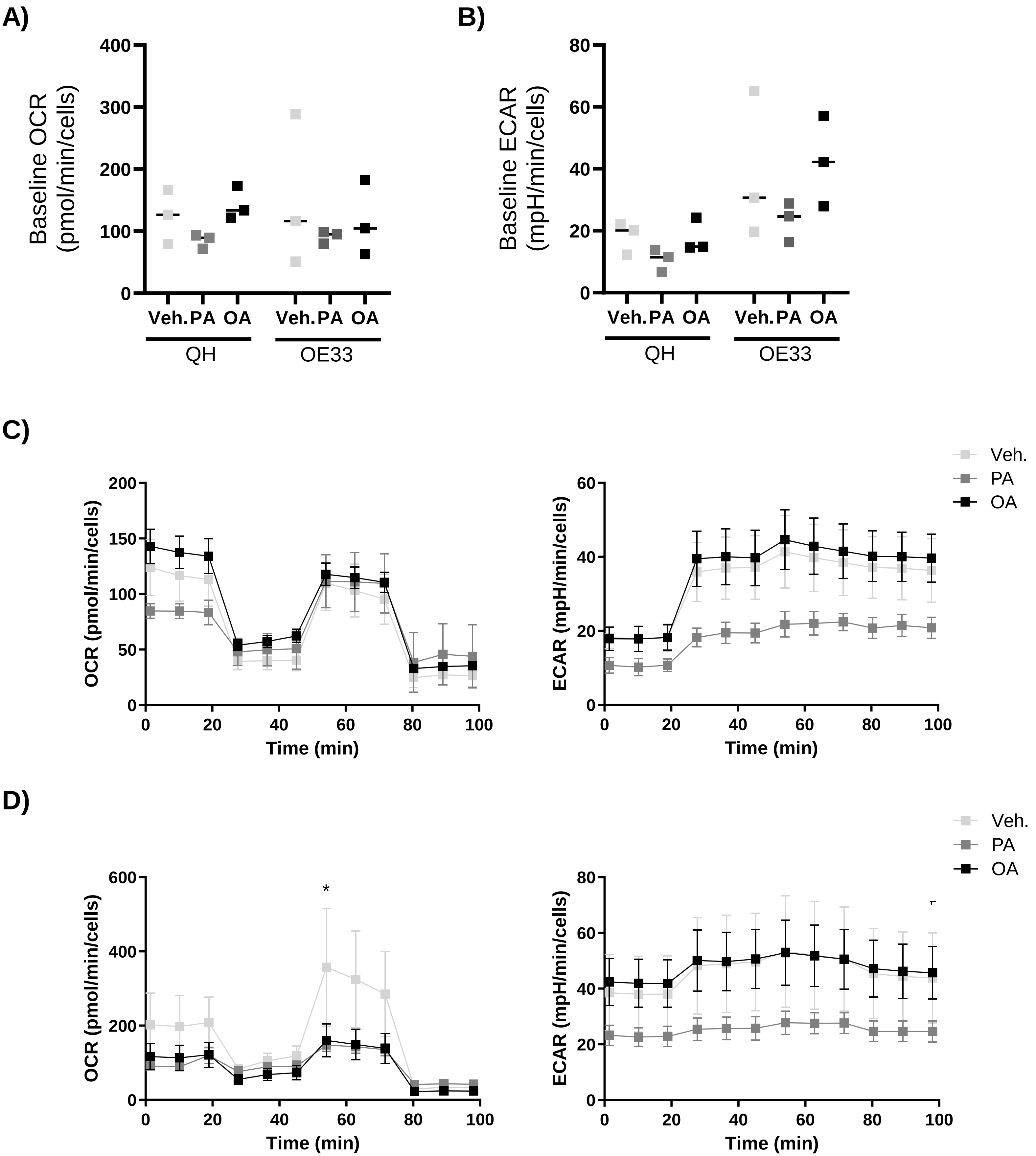

Supplement: Supplementary file 1 [file mmc1.pdf]
